# System Integration and Its Influence on the Quality of Life of Children with Complex Needs

**DOI:** 10.1155/2010/570209

**Published:** 2010-10-12

**Authors:** Sandy Thurston, Louise Paul, Chenglin Ye, Patricia Loney, Dillon Browne, Gina Browne, Maria Wong, Lehana Thabane, Peter Rosenbaum

**Affiliations:** ^1^Children's Treatment Network, Simcoe/York, ON, Canada L4M 2Y1; ^2^Research Unit on Health and Social Service Utilization, Faculty of Health Sciences, McMaster University, McMaster Innovation Park, 175 Longwood Road. S., Suite 210A, Hamilton, ON, Canada L8P 0A1; ^3^Department of Human Development and Applied Psychology, Ontario Institute for Studies in Education of the University of Toronto, ON, Canada M5S 1V6; ^4^Department of Clinical Epidemiology and Biostatistics, McMaster University, Hamilton, ON, Canada L8N 3Z5; ^5^Children's Health Research Institute, McMaster University, Hamilton, ON, Canada L8N 3Z5; ^6^CanChild Centre for Childhood Disability Research, McMaster University, Hamilton, ON, Canada L8S 4L8

## Abstract

*Purpose*. To explore the interactions between child and parents psychosocial factors and team integration variables that may explain improvements in physical dimensions of the PEDS QL quality of life of children with complex needs after 2 years. *Methods*. In this 2-year study, parents were identified by the Children's Treatment Network. Families were eligible if the child was aged 0–19 years, had physical limitations, resided in either Simcoe County or the Region of York, Ontario, and there were multiple other family needs. Regression analysis used to explore associations and interactions; *n* = 110. *Results*. A child's physical quality of life was affected by interacting factors including child's behavior, parenting, and integrated care. Statistically significant interactions between team integration, processes of care, and child/parent variables highlight the complexity of the rehabilitation approach in real-life situations. *Conclusions*. Rehabilitation providers working with children with complex needs and their families should also address child and parent problematic behaviors. When this was the case in high integrated teams, the child's physical quality of life improved after two years.

## 1. Introduction

Integration is the goal of many human service organizations, policymakers, theorists, and professionals. Integrated health services hold the promise of improved efficiency, capacity, performance/quality, cost-effectiveness, and enhanced working environment with improved communication and cooperation [1, 2]. The Canadian Council on Health Services Accreditation (2006) offers a definition of integration which encapsulates the many definitions found in the limited health literature “…*services, providers, and organizations from across the continuum working together so that services are complementary, coordinated, in a seamless unified system, with continuity for the client*” [3]. In Ontario, there are increasing efforts to promote health services integration especially in Local Health Integration Networks (LHINs). 

In a synthesis of reviews of effective, efficient human services for school-aged youth, Browne et al. [4] concluded that comprehensive interventions address multiple risk and protective factors, operate across multiple environments such as school, home, and community, and provide a mix of universal, targeted, and clinical services that are often proactive. This can be achieved by providing integrated care coordination (adjusting one provider's care because of what other providers do) or collaboration (jointly planning the type of care, provided how and by whom?). [1, 2] Recently the Children's Treatment Network (CTN) of Simcoe County and York Region, Ontario created integrated child and family teams of rehabilitation provider's from different and autonomous organizations whose providers work together using network interdisciplinary teams and a single plan of care to address the range and mix of any client's problems. Recently, the Children's Treatment Network (CTN) in Ontario was formed with funding from the Ontario Ministry of Children and Youth Services to create network interdisciplinary teams that target the needs of each child and their families with complex disabilities in Simcoe County and York Region of Ontario. The CTN model is a new approach for care of children and their families with complex needs that is based on local service providers from different agencies and organizations collaborating and taking a team approach to the comprehensive rehabilitation and psychosocial needs of both children and their families without the use of a single Children's Treatment Centre organization and building. 

The CTN model of team service provision consists of an “integrated package” with the following key elements.

- *Single point of access, Service Navigation,* and a comprehensive *Child and Family Interview, *
- assignment of a *Service Coordinator,* development of the individual *child and family team,* and development of a *single plan of care* recorded in the shared *electronic client record*.

This integrated network incorporates the key principles necessary for success as outlined by Suter et al. [3] in their comprehensive Health Systems Integration review. Common services available to families in the CTN model of delivery are developmentally appropriate physiotherapy, occupational therapy, speech therapy, augmentative communication, in-home family support worker, child behavior therapy, inclusive recreation, parenting instruction, family therapy, psychology, early childhood educator, or special educational resource teacher depending on the child's school age (http://www.ctn-simcoeyork.ca/).

To date, evaluation of health service integration efforts has been largely cross-sectoral in nature with some reported benefits to the system. System-level positive outcomes include reduction in nonemergency cases using the emergency room; reduction in the average length of stay in hospital; better financial performance; a flatter organizational structure (fewer management tiers). Some of the positive program/provider-level outcomes include increased job satisfaction; increased cooperation with other agencies; a blending of professional cultures into one shared culture [3]. There has been no measured link between integration efforts and patient outcomes. 

**Figure 1:**
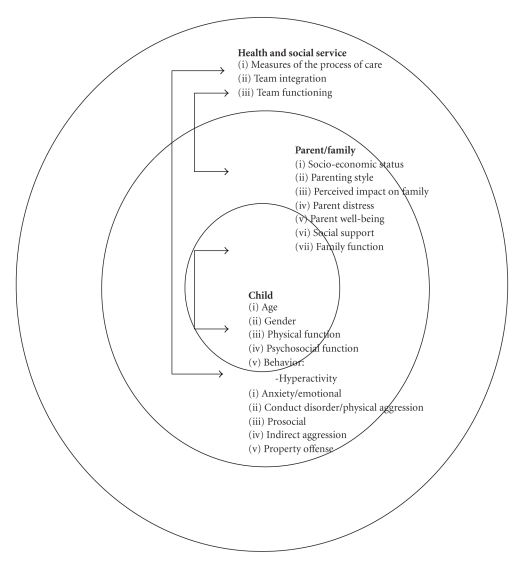
A visualization of interactions among levels of variables tested using Bronfenbrenner's Bio-ecological Model of Human Development.

Our team took the bioecological [5] and biopsychosocial [6] perspectives of human development that it is not possible to fully understand quality of life in children with complex needs by considering only simple associations between child/parent/family/social or health service provider factors. A multitude of levels of factors and interactions between these factors are simultaneously present as in Figure 1, and care of these families requires a holistic approach [7]. The purpose of this paper was to test the value of the integrated efforts of a team of rehabilitation providers from networked organizations that can be moderated by child and parent variables and relate these interactions to the change in the child's physical function after two years. 

We hypothesized that the effects of current integrated child and family team function would be more pronounced or stronger among families with fewer parent and child risk characteristics [6, 8] because the physical rehabilitation providers are not educated to deal with cumulative parent or child risk factors. When integrated teams are able to manage dysfunctional parents and child behaviors, there would be improvements in child physical function two years later. Parents and children interact and can moderate the effectiveness of integrated team function whereby either the child or the parent in interaction with service providers either facilitates or inhibits the success of physical rehabilitation efforts.

## 2. Methods

### 2.1. Research Setting

This descriptive study is part of a prospective cohort study examining the effects and expense of the CTN. Ethics approval was obtained for the study by the Research Ethics Board of McMaster University.

The depth of integration of CTN service providers on each child and family team was also measured at the 2-year followup. Completed integration measures were obtained from service providers on 110 CTN child and family teams.

#### 2.1.1. Study Design and Procedures

 This was a 2-year longitudinal survey of families with a special needs child enrolled in the CTN from May to December 2007. Families were deemed eligible if the child was aged 0–19 years, were residents of Simcoe/York, and there were, in addition to the child's physical rehabilitation needs, multiple other needs within the family (child's special cognitive, communication, and behavioral needs and families' needs e.g. parenting style, a parent's medical or mental health problem). The consenting parent/guardian most knowledgeable (PMK) returned a signed consent form to McMaster University indicating their willingness to participate. The PMK then completed a baseline telephone interview (1 hour) by one of three trained interviewers from McMaster University. A followup telephone interview was completed after 2 years by the same McMaster interviewer who was masked to the child and family receipt of integration care. 

### 2.2. Measures

#### 2.2.1. Human Service Integration

Integration of the CTN teams among families enrolled in this research was measured using the Integration of Human Services Measure [1, 2]. This measure evaluates observed and expected depth of integration among service providers on each CTN child and family team. Depth of integration represents the perceived and expected self-reported degree of exchange between all service providers along a continuum of involvement from nonawareness = 0, awareness = 1, communication = 2, cooperation = 3, collaboration = 4 [1]. The total *observed* integration score is the mean of the average group *observed* depth of integration scores. The total *expected* integration score is the mean of the average group expected depth of integration scores [1]. Scores range from 0 to 4. These scores represent the service providers' observed and expected levels of integration.

#### 2.2.2. Integrative Team Functioning

The team's functioning was assessed by the functioning of the service coordinator. Both observed and expected depth of integration scores about the service coordinator for each team were calculated by the average of individual members' inputs on a four-point scale from 0 (nonawareness) to 4 (collaboration). The number of team members who responded to the study and the corresponding response rate were also calculated.

#### 2.2.3. Child Quality of Life

The PedsQL is a generic measurement system developed by Varni et al. [9]. The shortened version consists of 15 items comprising three core scales and addresses the physical (5 items), emotional (4 items), social (3 items), and school functioning (3 items) [10]. Parent proxy report formats were used for children ages from 2 to 18 due to the inclusion of children with limited cognitive or communicative abilities. Each item for ages from 8 to 18 asks how much of a problem it has been during the past month on a five-point scale (0—“never a problem” to 4—“almost always a problem”). For children aged 5 to 7, the scale is modified to 0—“not a problem”, 2—“sometimes a problem,” and 4—“a lot of a problem.” Items are reverse-scored and linearly transformed to a 0–100 scale so that higher scores indicate better quality of life. Psychosocial Quality of Life (PsychQL) is computed as the sum of the Emotional, Social, and School scale scores (10 items, range 0–100). Reliability and validity of the shortened version have been documented [10].

#### 2.2.4. Child Behavior

Behavior was measured using the child behavior checklist (CBCL) questionnaire for children ages 2–19 also used in the Canadian Longitudinal Survey of Children and Youth (NLSCY) [11]. This allowed the comparison of this study sample with a nationally representative sample. The questionnaire asks about how the child seems to feel or act regarding age-specific behaviors such as getting into fights, inability to sit still, and worrying. The parent is asked to rate the specific behavior from 1—“never” to 3—“often”. Behavior subscales include hyperactivity/inattentive, prosocial, anxiety/emotional disorder, conduct disorder/ physical aggression, indirect aggression, and property offence. Items differ for age groups 0-1 years, 2–5 years, and 6–19 years. Specifically, questions pertaining to aggression, property offense, and prosocial behavior do not apply to the younger age groups. Internal consistency is reported by subscale and age group (Cronbach's alpha 0.68–0.84) [11, 12].

#### 2.2.5. Health of PMK

The Kessler scale (K10) [13, 14] measures PMK symptoms of depression and anxiety, a frequent accompaniment of depression. Ten questions measure the frequency of feeling: sad, nervous, restless, hopeless, worthless, everything was an effort, tired for no good reason, so nervous that nothing could calm down, fidgety, so restless could not sit still, or depressed during the past month. Chronic aspects of distress in the past month are examined on a five-point scale (1—“all of the time” to 5—“none of the time”). Reliability and validity have been documented [14]. Scores range from 10 to 50 where ≤19 indicates no clinically important level of distress, 20–24 indicates mild distress, 25–29 indicates moderate distress, and 30–50 severe distress.

#### 2.2.6. Parent Well-Being

 Parents were asked to rate their mental, physical health and general life satisfaction on a five-point scale (1—“very satisfied” or “excellent” to 5—“very dissatisfied” or “poor”). These questions were taken from the Canadian Community Health Survey (CCHS 2.2) [15] that allowed comparisons with national samples.

#### 2.2.7. Caregiver Burden

The Impact on Family (IOF) Scale determines the effects of a chronic illness on parents and families. Parents respond on a four-point scale about the degree to which statements apply to their family (1—“strongly agree” to 4—“strongly disagree”) [16]. The revised IOF scale (15 items) has been validated [17, 18]. Statements cover four dimensions: financial burden, family/social impact, personal strain, and mastery (e.g., fatigue is a problem, see family and friends less, need to change plans at last minute, little desire to go out).

#### 2.2.8. Parenting Practices

The NLSCY Parenting Scale [11] was used and consists of twenty-five questions adapted from the validated Parenting Practices Scale [19]. The following four parenting behaviors were measured: positive interaction (praise, play), hostility/ineffective (anger, ineffective discipline), consistency (follow through), and punitive (yelling, physical punishment). PMK rated each item (e.g., “Do something special with your child that he/she enjoys”) in terms of frequency from 0—“never” to 4—“many times each day.” Higher scores indicate greater frequencies for each type of parenting behavior. Internal consistency is reported by subscale and age group (Cronbach's alpha 0.39–0.75) [11].

#### 2.2.9. Social Support

The level of social support of the PMK was assessed using an eight-item shortened version of the Social Provisions Scale [20]. Different social support constructs were measured: guidance, reliable alliance (i.e., feeling assured that others would be available to offer practical help), and attachment. PMK rated each item along a four-point scale from 0—“strongly disagree” to 3—“strongly agree.” Higher scores represent greater social support. The reliability and validity of the measure have been reported [20].

#### 2.2.10. Family Functioning

Thirteen items from the NLSCY population survey [12], based on a subscale of the McMaster Family Assessment Device [21], were used to gather information on various aspects of family functioning (problem solving, communication, roles, affective responsiveness, affective involvement, behavior control). PMK rated each item (e.g., “We avoid discussing our fears or concerns”) along a four-point scale from 0—“strongly agree” to 3—“strongly disagree.” Negatively oriented items are reverse scored so that higher scores represent greater family dysfunction. The measure has good internal consistency (Cronbach's alpha 0.86) [21]. Scores range from 0 to 36 with scores ≥15 indicating family dysfunction.

#### 2.2.11. Parents' Perception of Family-Centeredness of Services

The Measure of Processes of Care (MPOC-20) is a 20-item, well-validated, and reliable self-report measure of parents' perceptions of the extent to which the services they and their child receive are family-centered [22, 23]. Respondents use a seven-point scale to describe the extent to which they experience service provider behaviors across five domains with response options ranging from 1—“never” to 7-“to a great extent.” The five domains are. Enabling and Partnerships, Providing General Information, Providing Specific Information, Comprehensive and Coordinated Care, and Respectful and Supportive Care. A “not applicable” category is included. MPOC scales have good internal consistency (Cronbach's alpha 0.77–0.96) [24].

#### 2.2.12. Child Demographics

They include, child age, gender, grade and PMK report of the main medical and other important diagnosis.

#### 2.2.13. Family Demographics

A standard form including spiritual or faith orientation, ethnicity, and languages was selected from the NLSCY that also includes community dwelling disabled children [12]. Sociodemographic data were gathered on the PMK gender, age, and educational level as well as on household income and family status.

## 3. Statistical Analysis

Descriptive statistics (numbers, percentages, means, and standard deviations) were calculated for all child and family variables, team integration, and team functioning scores. The child and PMK variables had a changing number of participants for several reasons. The behavior subscale measures have different numbers of items applicable to different age groups. Specifically, prosocial, indirect aggression, and property offence behavior scale items are applicable for children and youth from 6 to 19 years old. The PedsQL is applicable only to children aged 2–19. 

The behavior scales for different age groups were transformed using the interpolation technique where the mean of the behavior scale scores for children from 2 to 5 years old with fewer items were multiplied times the number of items for older children. This transformed mean was used in the analysis. In 18 instances, there were reports of two or three children with complex needs in the same family and only one report of parent variables. In these instances, the PMK was counted multiple times to ensure a matched number of children and parents in the analysis. 

A multiple linear regression model was used to study the interactions among the integrated team variables with other child/family/health services variables to explain the variation in the Child's Physical Quality of Life (QL) at followup. Variables consistent with the ecological conceptual framework [5] that showed high correlation with and strong prediction on the followup child's physical QL in the exploratory regression were selected. The selected child/family/health services/integration variables (i.e., independent variables) were then studied for all 2-way interactions with the outcome followup child physical QL (i.e., dependent variable). The fit of the model was assessed by the regression coefficient (*R* ^2^), as it measures the percentage of variation of the dependent variable explained by the model. In the final model, all possible 2-way interactions of variables were tested, and interactions that were not statically significant were removed using the *Forward Stepwise Selection* technique, where the inclusion significance level and exclusion significance level were chosen to be 0.05 and 0.10 respectively. The variables in the final model were centralized to adjust for possible multicolinearity. The normal probability plot for the residuals was used to check the normality assumptions for the models. 

The interactive effect between two continuous variables was illustrated by conditional regression lines. The association between one variable and the outcome was plotted as a regression line under three conditions of the other variable. The literature suggested applying one standard deviation from the mean to approximate scores of different conditions [25]. In our analysis, the three conditions were conventionally defined as high (any score more than 1 standard deviation above the mean), moderate (any score within 1 standard deviation of the mean), and low (any score more than 1 standard deviation below the mean). All analyses were performed using SPSS 15 (Chicago, IL).

## 4. Results

Table 1 shows the demographic characteristics of participating CTN families. The majority of PMK surveyed were mothers of the children (80%), born in Canada (74%), and spoke English (88%). The average PMK was 40 years, 86% were female (as 6% of the female respondents were not the child's mother), 84% were married/common-law, 69% were employed, and the median household income was $60–$69,000. There was an even split between families residing in Simcoe Region (50%) and York county. The average child age at interview was 7 years with 63% of the sample being male. Forty-four percent of the children were in pre-school (up to and including Kindergarten), 34% in grades 1–5 (elementary), and 22% in grades 6 to 12 (junior and high school). Fifty-seven percent of children were receiving service from Community Care Access Centers and School Boards at time of entry into the CTN. The top diagnoses for the children reported by PMK (Table 2) were mental and behavioral disorders (82%) including autism (29%), diseases of the nervous system (45%) including cerebral palsy (22%), and congenital malformations, deformations, and chromosomal abnormalities (26%). Fifty-four percent of children had more than one reported medical problem.

In Table 3, it can be seen that the CTN study sample included children with complex needs with very low physical and low psychosocial quality of life as indicated by the PMK compared to Varni's children and adolescents with other chronic diseases [26]. Generally, this sample of children exhibited prosocial behavior and low levels of anxiety, aggression, and property offence behaviors. PMK positive interaction and consistency parenting practices were moderate, and PMK hostile or ineffective parenting and punitive parenting were generally low in this sample. On average PMK report having social supports without family dysfunction and good overall life satisfaction. Forty-five percent, however, were exhibiting mild to severe symptoms of depression and anxiety (K10 > 19). For measures of processes of care, respectful and supportive care received the highest rating, and providing general information received the lowest rating by PMK. 

Observed levels of CTN team depth of integration indicate teams were currently functioning at a communication level while team members expect to be cooperating. The service coordinators were on average observed to be functioning at an awareness level and expected to be communicating. The average number of service providers on each child and family team was 6. The mean response rate per team was 67%. 

CTN teams were rated on their high (3.0 to 4.0) and low (<2.5) observed depth of team collaboration and expected team collaboration. Of 110 teams measured 43 were deemed high functioning (high observed and expected integration levels—bolded cells in Table 4). Sixty-seven teams were deemed low functioning based on low observed and expected integration scores (Table 4). A comparison of the children's characteristics, family functioning, and extent of health services received by families between the families engaged by high and low functioning teams is shown in Table 5. The difference in quality of life, although not statistically significant, is clinically important. Higher functioning teams were serving more physically and medically fragile functioning children with higher psychosocial quality of life, less aggression, and less prosocial behavior. In addition higher integrated teams were serving parents with more positive and less hostile parenting. Finally, the observed and expected service coordinator depth of integration scores were higher in teams scored as highly integrated.

**Figure 2:**
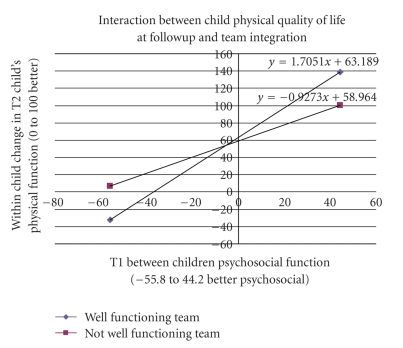


**Figure 3:**
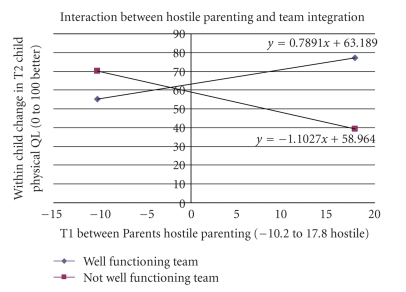


**Figure 4:**
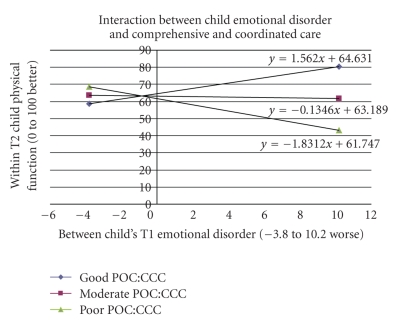


Table 6 presents the summary interaction regression analysis on the child's physical QL at followup. Eighty-nine percent of the variability in the child physical QL score at followup was explained by the model. A total of sixteen 2-way interactions among child/family/health services/integrative team variables were found to be statistically significant. Three tested the hypothesized health services/integration team variables interacting with child/family variables: child psychosocial QL interacting with integrated team function; child emotional disorder interacting with comprehensive and coordinated care; hostile parenting interacting with integrated team function. The interaction effect between two variables (e.g., *A* and *B*) was comprised of the main effects (*A* and *B*) and the cross effect (*A*∗*B*). Therefore, the regression coefficients of both components were included when interpreting a complete interaction effect. This could be easier to achieve by utilizing the plots of conditional regression lines. Figures 2–4 display the aforementioned three hypothesized interactions and show how integration was interacting with child/family variables. 

Child physical QL at followup was positively associated with child psychosocial QL at baseline (Figure 2). The strength of such association, however, interacted with the functioning of an integrative team. When the child and family team was highly by integrated, one additional unit of child psychosocial QL at baseline was associated with an average 1.7 score increase in child physical QL at followup. It was only associated with an average 0.9 score increase in child physical QL when the integrative team was low functioning. In other words, for each additional unit in children's psychosocial QL at baseline, there could be an average 82% improvement on the child's physical QL score at followup for children engaged by a high integrated team versus a low integrated team.

More hostile or ineffective parenting was not always positively associated with better child's physical QL (Figure 3). When the child and family team had low integration, for each unit increase in the baseline hostile ineffective parenting score on average, there was a deterioration of 1.1 in child's 2-year followup physical QL score. When the team was high functioning, there was on average a 0.79 improvement in the child's physical QL at followup. For each unit increase in hostile parenting at baseline, the high integration team effect could account for about 170% improvement in child physical QL at followup compared to the case of a similar situation receiving care from a low integrated team.

For each additional unit in child emotional disorder at baseline, there was an average 1.6 score increase in child physical QL at followup when the family received good comprehensive and coordinated care (Figure 4). In families receiving moderate and poor comprehensive and coordinated care at baseline, for each unit increase in child emotional disorder there were, on average, 0.13 and 1.83 decreases in child's physical QL scores at followup, respectively. The level of comprehensive and coordinated care received by the family interacted with the child's emotional status at baseline in its effect on the child's physical QL at followup. For families that received little to moderate comprehensive and coordinated care, the child's physical QL deteriorated at followup as their emotional status at baseline was worse. Contrarily, the child's physical QL improved at followup with poorer emotional function at baseline when the families received good comprehensive and coordinated support from service providers.

## 5. Discussion

This study provides original information about the effect of integrative efforts of individual child and family service teams for children with complex needs and reaffirms the value of a bioecological perspective. The primary hypothesis was corroborated about the effect of integrated team function on the improvement in child physical function after 2 years being more pronounced among families with fewer parent/child risk factors. Teams with higher integration scores worked with children with higher levels of psychosocial function and parents with more positive and less hostile ineffective parenting style at baseline. The physical function of these children improved more with the higher integrated team compared to the improvement in a similar child with less integrated team function. Further, when highly integrated teams worked with parents endorsing hostile-ineffective parenting styles at baseline, there was greater improvement in the child's physical function and quality of life two years later. A less integrated team working with parents with similar hostile-ineffective parenting styles at baseline resulted in the child's physical functioning actually deteriorating after 2 years. This same improvement in the child's physical function after 2 years was observed when parents of children with high emotional disorder at baseline reported receiving a high level of comprehensive coordinated care at followup compared to similar children receiving less comprehensive coordinated care. Generally, CTN child and family teams rated themselves as functioning at a communication level of integration. This combined with the response rate of “moderate” among service providers reflects the complexity of integrating service providers from different agencies all at differing locations. The CTN network is also still in its infancy with respect to organization, planning, and system support. Higher expected integration scores reflect the recognition from service providers of improving integration efforts over time. 

This study also provides information about the physical quality of life of children with complex needs and the associations and interactions of system integration variables. The low QL scores in this sample compared to others [26], particularly for physical quality of life, illustrate the multifaceted needs and issues faced by this heterogeneous group of children and youth with multiple diagnoses. 

This study supports previous findings and confirms that reports of simple associations between research outcomes do not give a comprehensive picture of the issues. Real-life problems are rarely caused by a single underlying issue. A multitude of factors (e.g., child behavior, child quality of life, parenting practices, provider processes of care, health service integration) and interactions among these factors are simultaneously present; therefore, care of these families requires a holistic approach that addresses all aspects of the child's environment. Finally, this study informs service planners of the positive characteristics of children and families more likely to be served by highly integrated teams, the risk variables at intake most likely to impair progress from children's rehabilitation services, and the need for teams to have behavioral mental health members.

## 6. Limitations

Results and findings are difficult to generalize outside of this study population because other contexts may differ. The PMK in this sample were predominantly married, educated, working mothers. This study may be missing important information from working, lower educated, single parents and their children—likely those with greater need. This study probably underestimates the effects. Highly integrated teams had already been working with a greater proportion of medically fragile children at intake into the study. While these families had less pronounced risk factors at intake into the study, this could have been due to the services of this team prior to the outset of the study.

Due to the cross-sectional nature of the team integration measure in of this research, we do not understand the causation or directional influence of integration on child quality of life. Longitudinal followup is needed to determine whether documented improvements in integrative team function can improve the well-being of the child or if in fact providers need to engage actively those difficult to reach families (i.e., families with low child psychosocial QL and unfavorable parenting practices). Of course randomized clinical trials would be the ideal design to address this question, but these are complex to undertake in circumstances like these [27]. These questions are also important in order that team integration efforts can be targeted and evaluated to include the needs of parents so that child quality of life can be maximized.

In this study, quality of life data were parent-reported. Generally, parents underestimate their child's quality of life compared to child self-reports [26]. Therefore, the associations and interactions may vary when child self-report data are used. It was not feasible to obtain self-report data from this complex needs group due to the wide range of limitations present in the children and budget constraints of the study. Finally, clinically important change was difficult to quantify in this patient population. Research to date has not determined the minimally important difference in quality of life in a diverse group of children with complex needs.

## 7. Conclusions

Rehabilitation providers working with children with complex needs need preparation to address child and parent problematic behaviors that limit progress in physical functioning. When this was the case in high integrated teams, the child's physical quality of life improved after two years.

## Figures and Tables

**Table 1:** Characteristics of sample (*n* = 110).

Variable		*n* = 110
*Respondent (PMK)*		
Age (years)	Mean (SD)	40.41 (6.90)
Gender	Female, *n* (%)	95 (86.4)
Relationship to child	Mother, *n* (%)	88 (80.0)
Marital Status	Married, *n* (%)	92 (83.6)
Employment status	Employed, *n* (%)	76 (69.1)
Country of birth	Canada, *n* (%)	81 (73.6)
Household language	English, *n* (%)	97 (88.2)
Household income	Median	$60–69,000
PMK Level of education	Median	Completed Postsecondary
PMK location of home	Simcoe, *n* (%)	55 (50.0%)

*Child *		
Age (years)	mean (SD)	7.36 (4.33)
Status	Preschool, *n* (%)	49 (44.5)
	Elementary, *n* (%)	37 (33.6)
	Junior, *n* (%)	24 (21.8)
Grade	Median	grade 1
Gender	Male, *n* (%)	69 (62.7)
Service provider	Early intervention, *n* (%)	40 (36.4)
	CCAC & School, *n* (%)	63 (57.2)
	New CTN referral, *n* (%)	7 (6.3)

PMK: Parent most knowledgeable

CCAC: Community Care Access Centre

CTN: Children's Treatment Network.

**Table 2:** PMK-reported child diagnosis (*n* = 110).

ICD-10 Diagnostic category		Count	%
A00–B99	Infectious and parasitic diseases	0	0.00
C00–D48	Neoplasm	1	0.91
D50–D89	Diseases of the blood and blood forming organs involving immune mechanism	0	0.00
E00–E90	Endocrine, nutritional, and metabolic diseases	4	3.40
F00–F99	Mental and behavioral disorders	90	81.82
F00–F84	Autism	32	29.09
F00–F84	Unspecified Disorder of psychological development	25	22.73
F00–F84	Specific developmental disorders of Speech and Language	10	11.00
F00–F84	Hyperkinetic disorders (ADD/ADHD)	13	9.09
G00–G99	Disease of Nervous system	49	44.55
	Cerebral Palsy	24	21.82
	Epilepsy	13	11.82
H00–H59	Disease of eye and adnexa	8	7.27
H60–H95	Disease of the ear and mastoid process	4	3.64
I00–I99	Disease of circulatory system	1	0.91
J00–J99	Diseases of respiratory system	4	3.64
K00–K93	Disease of digestive systems	0	0.00
L00–L99	Diseases of the skin and subcutaneous tissues	0	0.00
M00–M99	Diseases of the musculoskeletal system and connective tissues	2	1.82
N00–N99	Diseases of genitourinary system	2	1.82
P00–P99	Certain conditions originating in the perinatal period	1	0.91
Q00–Q99	Congenital malformations, deformations, and chromosomal abnormalities	29	26.36
	Down's syndrome	9	8.18

**Table 3:** Range, high score equivalency, and mean sample scores for measured variables.

Variables	*n*	Mean (SD)	Score Range	High Score Equivalency
*Child*				
Pediatric Quality of Life (age 2+ years)				
Physical function	103	46.93 (35.54)	0–100	Better function
Psychosocial function	103	58.16 (19.10)	0–100	Better function
Behaviour (age 2+ years)				
Hyperactivity/inattention	102	7.83 (3.93)	0–16	High activity/inattention
Anxiety/emotional	103	3.64 (2.95)	0–14	High emotional disorder
Conduct disorder/physical aggression	103	2.39 (2.66)	0–12	High conduct disorder
Prosocial	63	10.4 (6.04)	0–20	High prosocial behaviour
Indirect Aggression	63	0.95 (1.69)	0–10	High aggression
Property offence	63	1.38 (1.75)	0–12	High offence

*Family*				
Parenting				
Positive	109	15.71 (3.19)	0–20	More positive
Hostile	109	9.91 (5.29)	0–28	More hostility
Consistent	98	13.12 (3.92)	0–20	More consistency
Punitive	102	9.29 (2.05)	0–20	More punition
Social support	110	17.67 (4.60)	0–24	More support
Impact on family (score transformed)	110	22.37 (9.41)	0–45	Less adverse impact
Family function	110	9.15 (6.37)	0–36	High dysfunction
Parent distress(K10)	110	20.18 (5.95)	10–50	High distress
Parent report of life satisfaction	110	1.95 (0.90)	1–5	Poor life satisfaction
Parent report of mental health	110	2.37 (1.07)	1–5	Poor mental health
Parent report of physical health	110	2.48 (1.13)	1–5	Poor physical health

*Health Service*				
MPOC				
Respectful and supportive Care	110	5.10 (1.47)	1–7	Better perception
Providing general information	110	3.39 (1.54)	1–7	Better perception
Enabling and partnerships	110	4.57 (1.72)	1–7	Better perception
Providing specific information	110	4.93 (1.62)	1–7	Better perception
Comprehensive and cord. care	110	4.66 (1.66)	1–7	Better perception

*Integration*				
Team integration scores				
Observed depth	108	2.06 (0.69)	0–4	greater collaboration
Expected depth	108	2.74 (0.61)	0–4	greater collaboration
Team functioning scores				
Service coordinator observed	101	1.87 (1.18)	0–4	greater collaboration
Service coordinator expected	100	2.66 (0.95)	0–4	greater collaboration
Number of service providers	110	5.80 (2.46)	2–12	more team members
Response rate	110	67% (23%)	0–100	more response

**Table 4:** CTN team observed and expected integration levels.

			Mean expected integration score		Total
			Low (<3)	High (≥3.0)	

Mean observed integration score	Low (<2.5)	Count	67	**16**	83
		% of Total	60.90%	**14.50%**	75.50%
	High (>2.5)	Count	**3**	**24**	27
		% of Total	**2.70%**	**21.80%**	24.50%
	Total	Count	70	40	110
		% of Total	63.60%	36.40%	100.00%

**Table 5:** Comparison of followup characteristics between low and high team integration.

Variables (*n*)	Low Integration	High Integration	*t*-test	
	Mean (SD)	Mean (SD)	*t*	*P* value

*Child*				
Pediatric Quality of Life (age 2+ years)				
Physical function (103)	52.27 (35.05)	38.16 (32.23)	2.041	.044
Psychosocial function (103)	56.75 (18.23)	60.47 (20.47)	−0.957	.341
Behaviour (age 2+ years)				
Hyperactivity/inattention (102)	8.01 (3.8)	7.54 (4.17)	0.586	.559
Anxiety/emotional (103)	3.91 (2.95)	3.21 (2.93)	1.173	.243
Conduct disorder/physical aggression (103)	2.78 (2.8)	1.74 (2.27)	1.947	.054
Prosocial (63)	11.33 (5.53)	8.78 (6.67)	1.629	.109
Indirect aggression (63)	0.97 (1.63)	0.91 (1.83)	0.125	.901
Property offence (63)	1.58 (1.96)	1.04 (1.3)	1.161	.25

*Family*				
Parenting				
Positive (109)	15.19 (3.32)	16.52 (2.81)	−2.154	.033
Hostile (109)	10.67 (5.29)	8.69 (5.12)	1.921	.057
Consistent (98)	13.21 (3.86)	12.97 (4.07)	0.292	.771
Punitive (102)	9.36 (2.08)	9.19 (2.01)	0.41	.683
Social support (110)	17.82 (4.24)	17.44 (5.16)	0.42	.675
Impact on family (Score transformed) (110)	21.47 (8.74)	23.78 (10.32)	−1.262	.21
Family function (110)	9.61 (6.43)	8.42 (6.28)	0.958	.34
Parent distress (K10) (110)	20.35 (5.83)	19.92 (6.2)	0.367	.715
Parent report of life satisfaction (110)	1.97 (0.82)	1.91 (1.02)	0.342	.733
Parent report of mental health (110)	2.49 (1.08)	2.47 (1.22)	0.124	.902
Parent report of physical health (110)	2.37 (1.03)	2.37 (1.16)	0.005	.996

*Health Service-MPOC*				
Respectful and supportive care (110)	5.13 (1.42)	5.05 (1.56)	0.278	.782
Providing general information (110)	3.39 (1.6)	3.38 (1.47)	0.014	.989
Enabling and partnerships (110)	4.58 (1.76)	4.54 (1.67)	0.117	.907
Providing specific information (110)	5.14 (1.57)	4.61 (1.65)	1.682	.095
Comprehensive and cord. care (110)	4.66 (1.59)	4.66 (1.78)	0.004	.997

*Integration Team Scores*				
Observed depth (108)	1.72 (0.51)	2.57 (0.62)	−7.77	0
Expected depth (108)	2.37 (0.47)	3.31 (0.26)	−13.209	0

*Integration functioning scores*				
Service coordinator observed (102)	1.55 (1.05)	2.36 (1.21)	−3.537	.001
Service coordinator expected (101)	2.34 (0.94)	3.15 (0.76)	−4.545	0
Number of service providers (111)	5.99 (2.61)	5.51 (2.19)	0.988	.325
Response rate (111)	65% (23%)	72% (23.0%)	−1.579	.117

SD: Standard Deviation; *n*: sample size.

**Table 6:** Summary of regression analysis of final model predicting the child's followup physical function with only significant interactions.

Independent Variables	*Beta*	95% CI of *Beta *		*P*-value
High Functioning Team (yes = 1, no = 0)	4.22	−3.34	11.79	.278
Child Gender (male = 1, female = 0)	5.16	−0.96	11.29	.103
T1 Physical Function	0.94	0.84	1.04	<.0001
T1 Psychosocial Function	−0.91	−1.32	−0.49	<.0001
Child Hyperactivity	−0.88	−1.82	0.06	.070
Child Emotional Disorder	−0.13	−1.66	1.39	.863
Response Rate	0.27	0.08	0.46	.007
Group Observed Integration Mean	1.00	−5.18	7.18	.752
Hostile Parenting	−1.10	−2.17	−0.03	.047
Family Function	−0.01	−0.48	0.47	.982
MPOC: Respectful and Supportive Care	3.78	−0.67	8.23	.101
MPOC: Coordinated and Comprehensive Care	0.93	−2.41	4.26	.587
T1 Physical Function*	−0.04	−0.07	−0.01	.009
Child Hyperactivity				
T1 Physical Function*	0.20	0.03	0.37	.021
Group Observed Integration Mean				
T1 Physical Function*	0.03	0.01	0.06	.015
Hostile Parenting				
T1 Physical Function*	0.41	0.06	0.77	.026
Child Gender (male = 1, female = 0)				
T1 Physical Function*	−0.09	−0.14	−0.03	.005
Hostile Parenting				
T1 Physical Function*	0.27	0.11	0.44	.002
MPOC: Respectful and Supportive Care				
T1 Psychosocial Function*	0.77	0.34	1.20	.001
Well Functioning Team (yes = 1, no = 0)				
Child Hyperactivity*	1.02	0.32	1.72	.006
MPOC: Respectful and Supportive Care				
Child Emotional Disorder*	−0.62	−0.93	−0.31	.000
Hostile Parenting				
Child Emotional Disorder*	1.09	0.31	1.88	.008
MPOC: Coordinated and Comprehensive Care				
The % of Respondents on the Team*	−0.43	−0.71	−0.15	.003
Child's Gender (male = 1, female = 0)				
The % of Respondents on the Team*	0.04	0.00	0.08	.033
Hostile Parenting				
The % of Respondents on the Team*	0.17	0.09	0.25	<.0001
MPOC: Coordinated and Comprehensive Care				
Group Observed Integration Mean*	−1.87	−3.15	−0.60	.005
Hostile Parenting				
Hostile Parenting*	1.89	0.13	3.66	.040
Well Functioning Team (yes = 1, no = 0)				
MPOC: Respectful and Supportive Care*	−9.74	−14.51	−4.97	.000
Well Functioning Team (yes = 1, no = 0)				

*R-square = 0.8868

MPOC: Measures of Processes of Care, T1: Time 1.
